# Эндокринные и психосоматические нарушения у пациенток с аменореей

**DOI:** 10.14341/probl13366

**Published:** 2024-01-24

**Authors:** Ю. С. Абсатарова, Е. Н. Андреева, Ю. С. Евсеева, Т. А. Зеленкова-Захарчук, Е. В. Шереметьева, О. Р. Григорян, Р. К. Михеев

**Affiliations:** Национальный медицинский исследовательский центр эндокринологии; Национальный медицинский исследовательский центр эндокринологии; Московский государственный медико-стоматологический университет им. А.И. Евдокимова Министерства здравоохранения Российской Федерации; Национальный медицинский исследовательский центр эндокринологии; Национальный медицинский исследовательский центр эндокринологии; Национальный медицинский исследовательский центр эндокринологии; Национальный медицинский исследовательский центр эндокринологии; Национальный медицинский исследовательский центр эндокринологии

**Keywords:** гипогонадизм, синдром поликистозных яичников, гиперандрогения, функциональная гипоталамическая аменорея, преждевременная недостаточность яичников

## Abstract

В статье представлены данные о взаимосвязи патогенетических механизмов развития нарушений менструального цикла функционального и органического происхождения с психическими расстройствами с точки зрения психосоматической концепции. Согласно последней, функциональные нарушения менструального цикла рассматриваются как психосоматические, при которых гинекологическая патология развивается вследствие психопатологических расстройств. Ярким примером такого заболевания является функциональная гипоталамическая аменорея. При этом эндокринопатии, такие как синдром поликистозных яичников и преждевременная недостаточность яичников, также можно рассматривать в парадигме психосоматических нарушений овариальной функции, обусловленных высокой распространенностью тревожных и депрессивных расстройств в этой когорте больных. В данном обзоре подчеркивается важность междисциплинарного сотрудничества гинеколога и психиатра для максимально эффективной репродуктивной реабилитации пациенток с аменореей. Поиск литературы проводили в отечественных (eLibrary, CyberLeninka.ru) и международных (PubMed, Cochrane Library) базах данных на русском и английском языках. Приоритетным являлся свободный доступ к полному тексту статей. Выбор источников был приоритетен периодом с 2018 по 2023 гг. Однако с учетом недостаточной изученности выбранной темы выбор источников датировался с 1985 г.

## Abstract (en)

The article presents data on the relationship of pathogenetic mechanisms for the development of menstrual disorders of functional and organic origin in connection with mental disturbances from the point of view of the psychosomatic concept. According to the latter, functional disorders of the menstrual cycle are considered as psychosomatic, in which gynecological pathology develops as a result of psychopathological illness. A striking example of such a disorder is functional hypothalamic amenorrhea. At the same time, endocrinopathies, such as polycystic ovary syndrome and premature ovarian insufficiency, can also be considered in the paradigm of psychosomatic illnesses of ovarian function due to the high prevalence of anxiety and depressive disorders in this cohort of patients. This review highlights the importance of interdisciplinary collaboration between a gynecologist and a psychiatrist for the most effective reproductive rehabilitation of patients with amenorrhea. Literature search was carried out in national (eLibrary, CyberLeninka.ru) and international (PubMed, Cochrane Library) databases in Russian and English. The priority was free access to the full text of articles. The choice of sources was prioritized for the period from 2018 to 2023.However, taking into account the insufficient knowledge of the chosen topic, the choice of sources dates back to 1985.

## ВВЕДЕНИЕ

Аменорея — термин, обозначающий отсутствие менструаций у женщин. Это состояние может быть первичным или вторичным. Первичная аменорея — отсутствие менархе как такового, тогда как вторичная форма определяется как задержка менструаций более 3 мес при регулярном ритме менструаций и более 6 мес при нерегулярном цикле [1][2]. Данная патология может быть проявлением различных форм гипогонадизма: нормогонадотропного (например, синдром поликистозных яичников (СПЯ), врожденная дисфункция коры надпочечников), гипогонадотропного (функциональная гипоталамическая аменорея (ФГА), врожденный гипогонадотропный гипогонадизм) и гипергонадотропного (преждевременная недостаточность яичников — ПНЯ).

Психосоматические заболевания — это целый спектр патологических состояний междисциплинарного уровня, которые развиваются в результате двунаправленного взаимодействия психических/характерологических и соматических расстройств [3]. Так, известно, что патологические изменения уровней гормонов влияют на ментальность, а клинически завершенные тревожные и депрессивные расстройства могут сказываться негативно на функционировании репродуктивной системы. Таким образом, эндокринная психосоматика изучает взаимодействия гормональных нарушений и психопатологии, которые возникают под воздействием психосоциальных стрессов. Отдельно существует направление репродуктивной психосоматики, которая базируется на психосоматических соотношениях между нарушениями функции репродуктивной системы и психическими расстройствами. Психосоматические расстройства, по классификации академика РАН А.Б. Смулевича (2019), включают следующие категории:

ФГА — одно из самых очевидных психосоматических заболеваний в эндокринологии, представляющее собой гинекологическую патологию, обусловленную психическими расстройствами.

Взаимосвязь психофизиологических показателей и психопатологических нарушений, которые характерны для больных с гипоталамической аменореей, свидетельствует о вовлечении различных аспектов, связанных со стрессом, в патогенетические механизмы развития и прогрессирования заболевания. Поэтому функциональные изменения менструального цикла можно рассматривать как психосоматические расстройства [4]. Стоит отметить, что органическая патология (например, эндокринопатии СПЯ, ПНЯ) может провоцировать расстройства развития личности и психосексуального формирования, а психические расстройства, в частности протекающие с расстройствами пищевого поведения, могут приводить к манифестации эндокринных заболеваний, что создает специфические психосоматические соотношения у пациенток с аменореей.

Рассмотрим подробнее патогенез эндокринных нарушений при различных формах аменореи, а также особенности психического состояния больных в рамках психосоматической концепции.

## ФУНКЦИОНАЛЬНАЯ ГИПОТАЛАМИЧЕСКАЯ АМЕНОРЕЯ

ФГА — заболевание, которое часто встречается у пациенток на фоне снижения веса, чрезмерных физических нагрузок, психоэмоционального напряжения. Подавление репродуктивной функции в период «стресса» является естественным фундаментальным процессом выживания и способом защиты здоровья женщины с целью предотвращения беременности [5][6]. Американский колледж спортивной медицины даже выделил отдельный термин «триада спортсменок», который включает аменорею, расстройство пищевого поведения и низкую минеральную плотность костей [7].

На первой стадии заболевания при длительной задержке менструации в гормональном профиле пациенток можно обнаружить еще нормальные показатели гонадотропинов: лютеинизирующего (ЛГ) и фолликулостимулирующего гормонов (ФСГ), однако при продолжающемся воздействии стрессового фактора угнетение гипоталамо-гипофизарно-яичниковой оси приводит к снижению выброса гонадотропин-рилизинг-гормона (ГнРГ) и, соответственно, к низким уровням ЛГ, ФСГ и эстрадиола. Снижение пульсации ЛГ и низкий уровень эстрадиола впоследствии приводят к ановуляции и прекращению менструаций. Учитывая, что ФГА является вторичной формой аменореи, при которой нет структурно-органической причины, термин «функциональная» подчеркивает, что это состояние можно обратить вспять после выявления и устранения причины [8].

Психопатологические особенности пациенток с ФГА крайне важны в отношении развития, прогрессирования и общего прогноза заболевания [9]. Больные, страдающие функциональной аменореей, имеют значительно более высокие показатели депрессии и тревоги по сравнению с группой контроля, чаще проявляют дисфункциональные установки, такие как невротический перфекционизм с повышенной уязвимостью от мнения других людей, сообщают о внутреннем чувстве незащищенности и потребности контролировать малейшие изменения в жизни, им гораздо труднее адаптироваться к ежедневным событиям обыденной жизни по сравнению с женщинами без менструальных дисфункций [10][11]. Для пациенток с ФГА характерны также различные сексуальные нарушения, которые проявляются в виде снижения полового влечения, расстройства возбуждения, неадекватного увлажнения слизистых при половом акте, диспареунии [12]. В клинических исследованиях терапия эстрогенами уменьшала выраженность тревожных состояний у девочек с нервной анорексией и предотвращала их усиление по мере увеличения веса по сравнению с участницами, получавшими плацебо, что подтверждает важную роль эстрогенов как регуляторных гормонов в работе нейромедиаторов и нейротрансмиттеров, отвечающих за эмоции и настроение женщины [13].

Адекватный уровень эстрогена необходим и для полноценной реализации когнитивной функции. Эстрогендефицит негативно воздействовал на вербальную память и исполнительный контроль (термин используется для обозначения контроля моторной функции и когнитивных действий, направленных на достижение определенных целей) у спортсменок с олиго-аменореей по сравнению с атлетами с регулярным менструальным циклом и женщинами, не занимающимися спортом [14]. Когнитивные нарушения, которые регистрировали при манифестации ФГА у подростков и взрослых женщин, благополучно разрешались на фоне восстановления собственного ритма менструаций или при назначении заместительной гормональной терапии половыми стероидами [15].

Эстрогены оказывают влияние на многие области мозга: гипоталамус, мозжечок, нигростриарную и мезолимбическую системы, миндалевидное тело, гиппокамп, кору головного мозга и ствол мозга [13]. Сбои в работе нейротрансмиттеров серотонина, ацетилхолина, дофамина, норадреналина при эстрогендефиците могут усугубляться на фоне гиперкортизолемии, развивающейся при ФГА как ответ гипоталамо-гипофизарно-надпочечниковой системы на стресс. Дефицит эстрогенов и избыток кортизола, действуя синергически, формируют органическую основу для нейропсихических и нейрокогнитивных нарушений у пациенток с аменореей. Автономная нервная система у больных с ФГА, в свою очередь, запускает неадекватные вегетативные реакции (чрезмерное увеличение частоты сердечных сокращений, систолического и диастолического артериального давления) в ответ на действие «психологического раздражителя», т.е. восприятие стресса на физическом уровне при функциональной гипоэстрогении будет значительно отличаться от реакций здоровых женщин, а уровень кортизола при этом напрямую коррелирует с выраженностью тревожных, депрессивных расстройств и нарушениями пищевого поведения [16].

И хотя стрессовые факторы, которые могут привести к ФГА, изучаются давно, на данный момент не представляется возможным прогнозировать индивидуальную реакцию на них, или не всегда одинаковые тяжелые события вызывают сходные патологические реакции, что свидетельствует о генетической предрасположенности к менструальным дисфункциям, а стресс играет возможную роль триггера [17].

В клинических исследованиях была зарегистрирована значительная вариабельность возникновения нарушений менструального цикла после тяжелых тренировок. У спортсменок частота ФГА колеблется в пределах от 6 до 43%, в то время как у женщин, которые занимаются спортом любительски и выдерживают аналогичную схему тренировок, как у профессионалов, частота патологии значимо выше, и только у 14% женщин сохраняются регулярные менструальные циклы [18][19]. Изменчивость реакции гипоталамо-гипофизарно-яичниковой оси была подтверждена на экспериментальных моделях (макаках): выраженность овуляторной дисфункции зависела от степени стрессового воздействия [20]. Иммуногистохимический анализ срезов гипоталамуса устойчивых и чувствительных к стрессу обезьян выявил более высокую плотность нейронов, секретирующих ГнРГ, и более низкую плотность их волокон в срединном возвышении у чувствительных к стрессу животных, что говорит о различиях в нейронных механизмах, участвующих в синтезе, транспорте и высвобождении рилизинг-гормона в зависимости от стрессоустойчивости [21]. Данные работы показывают, что изменчивость генов, контролирующих развитие и функцию нейронов ГнРГ, может способствовать адаптации репродуктивной оси к стрессовым условиям. Эта гипотеза подтверждается недавними исследованиями, которые выявили более высокую частоту редких вариантов в генах, ассоциированных с идиопатическим гипогонадотропным гипогонадизмом, у пациенток с ФГА по сравнению с женщинами с регулярными менструациями [22]. Схожая клиническая картина этих двух патологических процессов, вероятно, обусловлена мутациями в генах, ответственных за развитие врожденного гипогонадизма, которые приводят к функциональному дефициту секреции ГнРГ, характерному для ФГА, в отличие от наследственной патологии, при которой полностью отсутствует пульсационная секреция рилизинг-гормона или нет его адекватного эффекта на гипофиз [23]. Кроме генетических вариантов и полиморфизмов существуют эпигенетические механизмы, которые регулируют работу гипоталамо-гипофизарно-яичниковой оси и, следовательно, принимают участие в развитии ФГА, формируя индивидуальную предрасположенность к ановуляции в результате стрессового события, или представляют собой биологические маркеры реакции на стресс, которые передаются как приобретенное привычное реагирование на прошлые значимые события [24].

Ведется поиск генов, ответственных за индивидуальную реакцию на стресс и предрасположенность к тревожным расстройствам. Специфические полиморфизмы (например, rs16147 и rs3214187) в нейропептиде Y, который выступает как регулятор энергетического баланса и противодействует анксиогенному эффекту кортикотропин-рилизинг-гормона, были связаны с устойчивостью или чувствительностью к стрессовым факторам [25]. Нейропептид Y контролирует концентрацию ГнРГ, индуцируя его высвобождение в присутствии адекватных уровней эстрадиола: при гипоэстрогении он подавляет выброс рилизинг-гормона, а при аменорее базальный уровень этого пептида резко снижается [26]. Таким образом, данный пептид, вероятно, выступает как связующее звено между осью гипоталамус-гипофиз-яичники и гипоталамус-гипофиз-надпочечники, а различные варианты в его гене могут влиять на чувствительность репродуктивной оси к стрессу.

Нейропластичность играет важную роль в реализации предрасположенности к расстройствам, ассоциированным со стрессом. Согласно нейротрофической гипотезе, аффективные нарушения могут быть связаны с изменением структурной пластичности и клеточной устойчивости, ключевую роль в которой играет мозговой нейротрофический фактор (BDNF). Последний активно экспрессируется в гиппокампе и участвует в нейрогенезе, нейропластичности, выживании нейронов и их дифференцировке. M. Miranda и соавт. обнаружили повышенные уровни BDNF в сыворотке при стрессовом воздействии и при аффективных расстройствах [27]. Несмотря на то что его роль в развитии ФГА до конца не ясна, у пациенток была зарегистрирована значительно более низкая концентрация этого фактора в плазме по сравнению со здоровыми женщинами, что, возможно, свидетельствует о вкладе BDNF в измененную реакцию на стресс при аменорее [28]. Эта гипотеза может стать основной для анализа распространенных вариантов гена BDNF, которые объяснили бы анксиогенный фенотип больных с ФГА.

Все больше работ описывают механизмы влияния на репродуктивную ось гипоталамус-гипофиз-яичники через систему кисспептинов, которая включает семейство гипоталамических нейропептидов, кодируемых геном KISS1 у человека [29]. Инактивация KISS1 и его рецептора, кодируемого KISS1R, приводит к задержке полового созревания и врожденному гипогонадотропному гипогонадизму [30], и, наоборот, активирующие варианты KISS1R и KISS1 вызывали центральное прежде­временное половое созревание [31]. Было обнаружено, что у пациенток с ФГА и ЛГ≤3 МЕ/л уровень кисспептина ниже по сравнению с участницами контрольной группы с ЛГ>3 МЕ/л (1,7 нг/мл и 2,6 нг/мл соответственно) [32]. Эти и многие другие работы легли в основу гипотезы о потенциальном благоприятном эффекте применения препаратов кисспептина для восстановления репродуктивной функции у женщин [33]. Первое применение этого экзогенного нейропептида у пациенток с ФГА было описано в работе С. Jayasena и соавт. (2009 г.) [34]. В рандомизированном контролируемом исследовании после однократного подкожного введения кисспептина наблюдалось 10-кратное увеличение секреции ЛГ и 2-кратное увеличение секреции ФСГ, а при длительном непрерывном введении в течение 2 нед подкожно развивалась тахифилаксия. В последующих работах были оптимизированы схемы введения препарата (дважды в неделю для пациенток с ФГА для стимулирующего эффекта на ЛГ и ФСГ, без серьезных побочных эффектов) [35], дозировки, которые восстанавливают как базальную, так и пульсационную секрецию ЛГ [36], а в 2020 г. был использован препарат агониста рецептора кисспептина, продемонстрировавший лучший профиль безопасности, стабильности и активности для больных с аменореей, по сравнению с собственно экзогенным гормоном [37].

Пока ведется разработка новых экспериментальных методик лечения ФГА, в клинической практике используются стандартные подходы, хорошо себя зарекомендовавшие, но не всегда дающие полное восстановление работы репродуктивной оси и излечение пациенток с гипогонадизмом. К методам лечения ФГА относят заместительную гормональную терапию половыми стероидами, психотерапевтическое лечение, фармакологическую терапию психотропными препаратами. На первом этапе адекватной стратегией при эстрогендефиците является обеспечение поступления экзогенных эстрогенов и прогестерона с целью поддержания костной системы и других органов и систем, где имеются эстрогеновые рецепторы [38]. Но всегда ли сработает одна только заместительная терапия? Учитывая установленную взаимосвязь между депрессивными расстройствами и нарушениями менструального цикла, патогенетически обоснованным подходом к ведению пациенток с ФГА является назначение антидепрессантов. Однако необходимо учитывать особенности различных фармакологических групп, так как некоторые препараты способны сами по себе вызвать менструальную дисфункцию [39]. При этом важно проводить дифференциальную диагностику между побочными эффектами, неэффективностью выбранной группы тимоаналептиков, некорректно подобранной дозировкой препаратов и ухудшением репродуктивной дисфункции или психического расстройства на фоне новых психосексуальных стрессов.

Трудности в лечении больных с аменореей заключаются не только в подборе адекватной схемы лечения, но и необходимости продолжать терапию после купирования основных жалоб и стабилизации менструального цикла для восстановления стрессоустойчивости. В российских и зарубежных рекомендациях по ведению пациенток с ФГА рекомендована психотерапия, в частности, когнитивно-поведенческая терапия (КПТ), направленная на улучшение способности справляться с психологическими стрессорами в будущем [1][2]. В среднем терапия занимает не менее 6 мес и не только способствует восстановлению овуляторной функции, но и снижает уровень кортизола — маркера гиперактивации гипоталамо-гипофизарно-надпочечниковой оси при ФГА [40]. КПТ может восстановить менструальный цикл, но если пациентка столкнется повторно со значимым стрессовым фактором, может случиться рецидив. Это обуславливает необходимость продолжения терапии антидепрессантами в течение 6–12 мес после нормализации менструальной функции.

Связь между психологическим стрессом и ФГА является двунаправленной, так как стресс подавляет ось «гипоталамус-гипофиз-яичники», и наоборот, низкий уровень эстрогена сильно влияет на психическое функционирование, что приводит к формированию порочного круга. Поэтому критически важным становится не только назначение адекватной заместительной гормональной терапии половыми стероидами, но и воздействие на психопатологическое звено болезни, включая психообразовательные стратегии (информирование пациентки об особенностях ее заболевания), в первую очередь со стороны гинеколога, а со стороны врача психиатра-психотерапевта — подбор психофармакотерапевтических препаратов для лечения тревожных, депрессивных расстройств и проведения психотерапии для корректирования патологических когнитивных и поведенческих искажений. Эффект от них будет максимальным только при сочетании всех перечисленных методик.

## СИНДРОМ ПОЛИКИСТОЗНЫХ ЯИЧНИКОВ

СПЯ и ФГА имеют схожую клиническую картину, поэтому их дифференциальная диагностика представляет некоторые трудности. Нарушения менструального цикла вплоть до аменореи, редкие овуляции, мультифолликулярные или поликистозные яичники при ультра­звуковом исследовании могут встречаться одинаково часто в обоих случаях, хотя патофизиология этих синдромов совершенно различна. До настоящего времени используют роттердамские критерии для постановки диагноза СПЯ, предложенные еще в 2003 г. [41]. В основе патогенеза этого заболевания лежат гиперандрогения и инсулинорезистентность, однако этиология его еще до конца неизвестна. Можно ли считать данный синдром психосоматическим заболеванием в классическом понимании? Вероятно, гиперандрогения будет той самой «­соматической» почвой, на которую наслаиваются личностные особенности, и тогда мы говорим о высокой распространенности тревожно-депрессивных расстройств, которые развиваются на фоне клинических проявлений избытка андрогенов, ожирения и бесплодия. Но что, если после устранения косметических дефектов, стабилизации менструальной функции и снижения веса не удается поддержать стойкую овуляцию? Возможно ли, что рецидив СПЯ связан с отсутствием коррекции психического статуса и для длительной ремиссии требуется адекватный показатель ментального здоровья пациентки с учетом хронического течения заболевания? На эти вопросы пока нет однозначного ответа.

Известно, что среди больных СПЯ отмечается высокая распространенность различных пограничных психопатологических нарушений, таких как депрессивные, обсессивно-компульсивные нарушения, расстройства личности, генерализованное тревожное расстройство, социальные фобии, синдром дефицита внимания и гиперактивности, а также расстройства пищевого поведения. Расстройства психотического регистра (биполярные аффективные расстройства, шизофрения и другие) также чаще, чем в общей популяции, диагностируют у женщин с овариальной гиперандрогенией. Кроме того, высокая частота психопатологий может быть еще обусловлена как собственно гиперандрогенией, так и вторичными депрессивными и тревожными расстройствами, возникающими вследствие развития андрогенной дерматопатии (акне, гирсутизм, алопеция), ожирения, бесплодия, что, несомненно, стигматизирует женщин и снижает качество их жизни [42]. Активно изучается вопрос влияния хронического воспаления на развитие депрессивных расстройств, в том числе при СПЯ [43]. Психопатология может усугублять течение данного синдрома и затруднять лечение, особенно это касается ановуляторного бесплодия, которое иногда с трудом поддается коррекции даже с использованием вспомогательных репродуктивных технологий [44].

Интересен тот факт, что среди братьев и сестер больных СПЯ отмечают такой же высокий риск развития различных психических расстройств. Источником проблемы, по-видимому, является неблагоприятное воздействие андрогенов на формирование мозга во внутриутробном периоде. Возможно, гиперандрогения вызывает изменения в его структуре, что приводит к аномальным реакциям на стероидные гормоны. На экспериментальных моделях было показано, что при избытке мужских гормонов развивается так называемая «маскулинизация» мозга, которая способствует программированию соответствующих паттернов поведения в дальнейшем [45]. Возможно, раннее воздействие андрогенов на рецепторы в этом органе может необратимо его реорганизовать и привести к гиперактивности нейронов ГнРГ во взрослой жизни, что в итоге стимулирует избыточную секрецию ЛГ [46]. И тогда пациентка, генетически предрасположенная к определенным физиологическим процессам, в течение жизни реализует запрограммированный «андрогенный» сценарий.

Учитывая хроническое течение СПЯ, важным аспектом ведения женщин репродуктивного возраста является оценка качества жизни и психосоциального воздействия заболевания [47]. A. Rempert и соавт. проанализировали данные 14 рандомизированных контролируемых и 19 наблюдательных исследований, в которых изучили показатели качества жизни с помощью стандартизированных опросников до и после лечения синдрома, и сравнили с аналогичными значениями в контрольной группе [48]. Авторы заключили, что СПЯ по воздействию на качество жизни сопоставим и даже превосходит болезни сердца, сахарный диабет и рак молочной железы. После лечения улучшаются показатели, связанные с психическим здоровьем, бесплодием, сексуальной дисфункцией, ожирением, нарушением менструального цикла и гирсутизмом.

В недавней работе А. Adamczak и соавт. оценили опосредованное влияние временнóй перспективы на развитие депрессивных симптомов у пациенток с СПЯ с помощью стандартизированных опросников (шкала депрессии Бека BDI–II и опросник Зимбардо ZTPI) [49]. Временнáя перспектива — это совокупность представлений человека о своем психологическом будущем и прошлом, существующих в данный момент времени. Этот термин используют для описания позитивного и негативного отношения индивидуума к прошлому и будущему, а также гедонистического и фаталистического отношения к настоящему. Например, негативный взгляд на прошлое может сформировать пессимистическое и депрессивное отношение к настоящему или будущему. Исследователи заметили, что изменение параметров временной перспективы часто связано с развитием депрессивных симптомов. Опросник Зимбардо ZTPI позволяет проанализировать состояние пациенток по 4 шкалам: шкала положительного прошлого оценивает концентрацию на положительном прошлом; шкала негативного прошлого — концентрацию на негативном прошлом, т. е. размышлениях о неприятных воспоминаниях и неудачах; шкала гедонистического настоящего относится к сосредоточенности на сиюминутных удовольствиях независимо от последствий своих импульсивных действий; шкала будущего измеряет нацеленность на будущее, т.е. направленность на планирование, успех и последовательное осуществление своих жизненных целей. В исследовании с участием 83 больных и 65 здоровых женщин было обнаружено косвенное влияние депрессивных симптомов на СПЯ через позитивную перспективу будущего. Временная перспектива связана с психосоматическими симптомами синдрома, качеством жизни, стрессом и формированием ответственного поведения в отношении здоровья. Позитивная перспектива будущего влияет на осмысление молодой женщиной личных потребностей через осознание своей психосексуальности, в том числе благополучную реализацию репродуктивной функции (половая жизнь, создание семьи, рождение детей). Депрессивный настрой связан с тревогой ожидания будущего, негативным восприятием потенциальных партнеров, избегающим поведением в построении близких отношений из-за страхов ошибиться, разочароваться, получить душевную боль. Вероятно, негативное отношение к необходимости реализации в будущем личной жизни актуализирует психосоматические соотношения, тесно связанные с нарушениями психосексуального самовосприятия, отрицательно влияет на реализацию возможности привлечения благополучного партнера и тем самым угнетающе действует на репродуктивную систему. Более того, клинические проявления гиперандрогении (акне, гирсутизм, алопеция) заставляют пациенток чувствовать себя менее привлекательными, что негативно сказывается на их психическом здоровье из-за снижения самооценки и физической удовлетворенности [50]. Работа междисциплинарной команды, включающей психотерапевта, клинического психолога, может помочь женщинам поддерживать самодостаточность и возможность повлиять на жизненную ситуацию, таким образом, подключение образовательных и терапевтических психотренингов является профилактическим мероприятием в развитии депрессивных расстройств, связанных с СПЯ, и повышает стрессоустойчивость.

Одним из подходов, который может улучшить психическое здоровье и качество жизни пациенток с овариальной гиперандрогенией, является КПТ. Оценка ее влияния на тревогу, депрессию и качество жизни была проведена S. Majidzadeh и соавт. среди 84 больных СПЯ, рандомизированных в основную группу и группу контроля [51]. Консультирование проводилось по 60–90 мин, всего 8 сеансов, еженедельно в группах по 5–7 человек, анализ психического статуса проведен с помощью опросников Бека и анкеты качества жизни PCOSQ. В группе КПТ показатели тревоги и депрессии оказались значимо ниже по сравнению с контролем, а средний балл качества жизни — выше. Подтверждение эффективности психотерапевтических методик у больных СПЯ продемонстрировали H. Dema и соавт., более того, они обнаружили динамические изменения в метилировании ДНК в периферической крови 4 генов: COMT, FST, FKBP51 и MAOA [52]. Эпигенетические изменения, такие как метилирование ДНК, могут играть роль в развитии и прогрессировании патологической функции яичников и метаболических нарушений при овариальной гиперандрогении. Вероятно, эти эпигенетические процессы являются ответом на различные вмешательства, направленные на изменение факторов здоровья и образа жизни.

Конечно, нельзя игнорировать тот факт, что нефармакологические методы воздействия (модификация образа жизни, соблюдение принципов рационального питания и дозированные физические нагрузки) в большинстве случаев уже помогают контролировать течение СПЯ, особенно у пациенток с метаболическим фенотипом. Физические упражнения могут положительно сказываться не только на метаболических показателях, но и на ментальном здоровье больных, что было продемонстрировано I. Santos и соавт. в рандомизированном клиническом исследовании [53]. 23 молодых женщины с СПЯ были случайно распределены на 2 группы: в основной группе участницы занимались интенсивной физической активностью по 40–60 мин в день, 3 дня в неделю, в течение 12 нед с последующим полным прекращением тренировок в течение 30 дней, в группе контроля пациентки не занимались спортом. Оценку ментального здоровья провели с помощью опросника SF-36 и шкалы депрессии, тревоги и стресса (DASS-21) в 3 точках: 1) исходно; 2) после 12 нед занятий спортом; 3) после 30 дней отсутствия тренировок. В основной группе на протяжении всего периода нагрузок пациентки отмечали значимое улучшение показателей качества жизни: физических возможностей и общего восприятия здоровья, при этом выраженность симптомов тревоги и депрессии значительно уменьшилась, но через 30 дней отсутствия тренировок показатели изменились в худшую сторону, а психический статус был крайне нестабильным. Из этого следует, что, когда практика физических нагрузок прерывается или прекращается, в организме нивелируются положительные адаптивные изменения уже через 4 нед. Это еще раз подтверждает необходимость соблюдения принципов поддержания здоровья пациентками с СПЯ на протяжении всей жизни.

В похожем исследовании R. Patten и соавт. пошли дальше и попытались выяснить, какая интенсивность нагрузки обеспечит лучший контроль психологических и физических показателей при овариальной гиперандрогении и избыточной массе тела у женщин с СПЯ [54]. Авторы сравнили интервальную тренировку высокой интенсивности и стандартную непрерывную тренировку средней интенсивности и обнаружили, что в первом случае значимо снизились показатели депрессии, тревоги и стресса, полученные с помощью опросников, а во втором — только показатели стресса. Это исследование подчеркивает потенциал высокоинтенсивных занятий спортом для улучшения психического здоровья и качества жизни и может быть оптимальной стратегией для уменьшения симптомов депрессии и тревоги.

Почему физические нагрузки приводят к столь значимым изменениям? Известна ассоциация между инсулинорезистентностью, метаболическим синдромом и риском депрессии при СПЯ [55]. Вероятно, на фоне тренировок снижаются гиперинсулинемия и инсулинорезистентность, повышается уровень секс-стероид-связывающего глобулина, «утилизируется» избыток андрогенов, т.е. нейтрализуются 2 основных патогенетических звена заболевания и, как следствие, восстанавливается менструальная и овуляторная функция, а пациентка становится более удовлетворенной своим внешним видом. Дополнительный вклад может оказывать серотонин, который усиленно вырабатывается при занятиях спортом [56].

С другой стороны, нет данных, как чрезмерные физические нагрузки в долговременной перспективе сказываются на психопатологических расстройствах, которые манифестировали до начала тренировок. Ведь, как известно, при ангедонии, апатии нет желания и сил заниматься физической активностью, именно поэтому могут нарушаться рекомендации соблюдать режим активных тренировок и здоровый образ жизни, и без лечения депрессивных расстройств это окажется малоэффективным. Кроме того, при длительной детренированности активные занятия спортом могут усугубить соматическое состояние в силу наличия сопутствующих ограничивающих заболеваний, и достичь высокого уровня тренированности будет проблематично.

Тогда коррекция психического статуса должна проводиться с использованием и психотерапии, и психофармакологической поддержки, что в теории должно восстановить менструально-овуляторную функцию и повысить стрессоустойчивость. На экспериментальных моделях было показано, что назначение амитриптилина — трициклического антидепрессанта улучшало морфологию и функцию яичников, а также эстральный цикл при СПЯ, индуцированном эстрадиола валератом. Антидепрессант восстанавливал уровни эстрадиола, тестостерона и прогестерона до нормы и повышал уровень ЛГ, хотя влияния на ФСГ не было зарегистрировано [57]. Похожее влияние амитриптилина на поликистозную структуру яичников было установлено в работах других авторов [58]. Хотя, как известно, сам амитриптилин необходимо назначать с осторожностью пациенткам с избыточной массой тела, так как в ряде случаев он может повышать аппетит и, следовательно, увеличивать вес. В связи с чем предпочтительнее выбирать тимоаналептики двойного и тройного действия современных поколений.

Таким образом, терапия СПЯ не должна ограничиваться только назначением гормональных препаратов; обязательным условием является включение в команду специалистов врача-психотерапевта и клинического психолога для оптимальной психофармакологической поддержки и психотерапевтической коррекции патологически искаженных когниций пациентки в процессе психотерапии с целью повысить самооценку, улучшить качество жизни и нормализовать репродуктивную функцию.

## ПРЕЖДЕВРЕМЕННАЯ НЕДОСТАТОЧНОСТЬ ЯИЧНИКОВ

ПНЯ — заболевание, при котором у женщин до 40 лет прекращается функция яичников, что сопровождается повышением уровня гонадотропинов, снижением уровня эстрогенов и в итоге приводит к аменорее, бесплодию и отдаленным последствиям для здоровья: раннему развитию сердечно-сосудистых заболеваний, остеопорозу, снижению когнитивных функций и общей ожидаемой продолжительности жизни. Для постановки диагноза требуется зарегистрировать повышение ФСГ более 25 МЕ/л дважды с разницей в месяц, можно обнаружить снижение количества антральных фолликулов по УЗИ, а также уровня антимюллерова гормона и эстрадиола [59]. Распространенность этой патологии составляет в среднем 3,5% в популяции, при этом в развивающихся странах она немного выше, чем в развитых, — 5,1%. Вероятно, это связано с худшими социально-экономическими условиями, состоянием индивидуального и общественного здоровья, затрудненным доступом к профилактическим медицинским услугам или информации [60].

Основные причины ПНЯ — это генетические аномалии, аутоиммунные процессы, тяжелые инфекции, ятрогенные факторы (хирургические вмешательства, лучевая/химиотерапия), семейные формы заболевания и идиопатические причины [59]. Среди генетических факторов наиболее часто встречаются хромосомные патологии (синдром ломкой Х-хромосомы), дисгенезия гонад, синдром Шерешевского–Тернера. Однако в некоторых случаях у пациенток с ПНЯ обнаруживают аномальные эпигенетические изменения. К последним относятся метилирование ДНК, экспрессия некодирующих РНК и модификации гистонов. Эти процессы выполняют регулирующую функцию в отношении экспрессии генов и служат одним из ключевых факторов, влияющих на микроокружение яичников и развитие фолликулов, тогда как их аномалии нарушают созревание ооцитов [61]. Аутоиммунная патология как причина ПНЯ составляет 10,5% от общего числа случаев истощения яичников и включает аутоиммунные эндокринные (надпочечниковая недостаточность, тиреоидит Хашимото и сахарный диабет 1-го типа) и неэндокринные расстройства (синдром Шегрена, ревматоидный артрит, системная красная волчанка, воспалительное заболевание кишечника, рассеянный склероз, тяжелая миастения) [59]. Однако более чем в 50% случаев этиология заболевания неизвестна даже после тщательного обследования — это спонтанная или идиопатическая форма данной нозологии. Возможно ли, что именно психопатологические расстройства в этом случае будут пусковым фактором раннего истощения овариального резерва, и ПНЯ является классическим психосоматическим заболеванием, которое реализуется при наличии генетической предрасположенности или аномальных эпигенетических изменений у женщины?

Известно, что среди пациенток с ПНЯ высок риск депрессивных и тревожных расстройств. По данным метаанализа, куда были включены 1316 участниц, отношение шансов (ОШ) для депрессии составило 3,33, 95% доверительный интервал (ДИ)=2,31–4,81, р<0,001; для тревоги — ОШ=4,89, 95% ДИ=3,28–7,30, р<0,001 [62]. Психические расстройства у женщин с ранним истощением овариального резерва часто остаются недиагностированными. Пациентки обычно обращаются по поводу бесплодия и проблем с менструальным циклом, игнорируя ментальные проблемы. К сожалению, иногда врачи склонны не признавать значимость психосоматических проблем этой когорты больных, что приводит к тому, что у трети из них пропускают диагноз тревожных или депрессивных расстройств средней или тяжелой степени. Психопатологические нарушения могут, в том числе, повлиять на фертильность и результаты лечения бесплодия. J. Ventura и соавт. обнаружили положительную корреляцию между психическим статусом и овариальной функцией у пациенток с ПНЯ [63]. Психосоциальное влияние этой нозологии может затронуть и партнеров-мужчин, что приводит к неадекватному восприятию болезни их жен. K. Chu и соавт. проанализировали показатели тревоги, депрессии и супружеских отношений у 52 мужчин в основной группе (ПНЯ) и 52 участников контрольной группы [64]. Мужчины из 1-й группы испытывали повышенный уровень тревоги и депрессии, а также меньше были удовлетворены супружескими отношениями в некоторых аспектах по сравнению с группой контроля. Многие партнеры не имели достоверной информации о заболевании их спутниц, что может быть результатом недостаточного консультирования со стороны врачей. Более того, их уровень понимания болезни коррелировал с тревогой (r=-0,64; р<0,01), депрессией (r=-0,38; р<0,01) и отношениями в общем (r=0,28; р<0,05). Таким образом, пациентка может испытывать дополнительную психоэмоциональную нагрузку в браке, что будет усугублять нарушения ее психического состояния. При этом не указывается, находились ли супруги пациенток с ПНЯ в тревожно-депрессивном статусе до брака, может, причина в искаженном выборе самих женщин в состоянии психического истощения.

Точная этиология тревожных и депрессивных расстройств у пациенток с ПНЯ пока неизвестна. Вероятно, здесь задействованы механизмы нейроэндокринной регуляции в оси гипоталамус-гипофиз-яичники, цитокины и психосоциальные стрессы. Избыточный выброс ФСГ и снижение уровня эстрогенов непосредственно влияют на синтез, метаболизм и рецепторную активность в системе нейротрансмиттеров: 5-гидрокситриптамина, дофамина и норадреналина, что в итоге повышает риск развития депрессивных и тревожных нарушений у женщин в менопаузе [65]. Аналогичный процесс, возможно, идет и у больных ПНЯ. Еще один механизм патогенеза психопатологических расстройств может быть связан с уровнем трансформирующего фактора роста-β и гамма-интерферона. Была обнаружена их ассоциация с развитием раннего истощения овариального резерва и депрессивных расстройств, что можно объяснить иммунными нарушениями и активацией системы воспалительного ответа [66]. Другой немаловажной причиной аффективной патологии является психосоциальный стресс, который непосредственно взаимосвязан с инфертильностью пациенток. У женщин с бесплодием значимо чаще выявляют расстройства настроения, а у больных ПНЯ ситуация усугубляется тем, что манифестация преждевременного прекращения овариальной функции может произойти в очень молодом возрасте, когда пациентка даже не задумывалась о своих репродуктивных планах, что приводит к еще большему уровню стресса, усиливающегося на фоне общественной стигматизации инфертильности [67][68]. Не стоит забывать о том, что психопатологические расстройства могут инициировать развитие аменореи и ухудшать овариальную функцию, как было рассмотрено выше (ФГА). Хронические негативные эмоции активируют ось гипоталамус-гипофиз-надпочечники, что приводит к секреции большого количества кортизола, который подавляет пульсирующий ритм высвобождения ГнРГ [69]. Кроме того, психический стресс индуцирует синтез β-эндорфина в гипоталамусе и гипофизе, опосредованно влияя на выброс гонадотропинов, а те, в свою очередь, — на функцию яичников, что может привести к развитию ПНЯ. Поэтому на вопрос, являются ли психопатологические нарушения преморбидным фоном для идиопатической («психогенной») формы ПНЯ или сопутствуют начинающемуся эстроген-дефициту, нет однозначного ответа.

## ЗАКЛЮЧЕНИЕ

Психосоциальные стрессы имеют множество неблагоприятных последствий для здоровья. Оценить негативный эффект пролонгированной психогении проблематично, так как отсутствуют объективные критерии. У женщин фенотипические маркеры хронического стресса включают нарушения менструального цикла, аменорею или бесплодие. Редкие полиморфизмы, которые контролируют развитие и/или функцию нейронов ГнРГ, могут способствовать приспособляемости репродуктивной оси к стрессовым факторам. ФГА является классическим психосоматическим заболеванием, когда соматическая болезнь провоцируется (обусловлена) психической патологией, расстройствами личности или психогенными факторами. При этом сопутствующие депрессивные и тревожные расстройства, часто диагностируемые у пациенток с СПЯ и ПНЯ, могут выступать как пусковой фактор в манифестации эндокринопатии, что позволяет отнести данные заболевания к психосоматическим. Поэтому междисциплинарная команда гинеколога и психиатра сможет улучшить индивидуальный терапевтический подход, основанный на таргетной терапии, и обеспечит репродуктивную реабилитацию пациенток с различными формами аменореи.

## ДОПОЛНИТЕЛЬНАЯ ИНФОРМАЦИЯ

Источники финансирования. Работа выполнена в рамках государственного задания №123021300169-4 «Эпигенетические предикторы и метаболомная составляющая аменореи различного генеза у женщин репродуктивного возраста», 2023–2025 гг.

Конфликт интересов. Авторы декларируют отсутствие явных и потенциальных конфликтов интересов, связанных с содержанием настоящей статьи.

Участие авторов. Все авторы одобрили финальную версию статьи перед публикацией, выразили согласие нести ответственность за все аспекты работы, подразумевающую надлежащее изучение и решение вопросов, связанных с точностью или добросовестностью любой части работы.

## References

[cit1] Rossiiskoe obshchestvo akusherov-ginekologov. Klinicheskie rekomendatsii. Amenoreya. — M.: Ministerstvo zdravookhraneniya RF; 2021.

[cit2] Gordon CM, Ackerman KE, Berga SL, et al. Functional hypothalamic amenorrhea: An endocrine society clinical practice guideline. J Clin Endocrinol Metab. 2017;102(5):1413-1439. doi: https://doi.org/10.1210/jc.2017-00131 28368518

[cit3] Psikhosomaticheskie rasstroistva v klinicheskoi praktike, 2-e izd / Pod red. Smulevicha A.B. — M.: MEDpress-inform; 2019.

[cit4] Berga Sarah L, Marcus Marsha D, Loucks Tammy L, Hlastala Stefanie, Ringham Rebecca, Krohn Marijane A (2003). Recovery of ovarian activity in women with functional hypothalamic amenorrhea who were treated with cognitive behavior therapy. Fertility and Sterility.

[cit5] Volel BA, Ragimova AA, Burchakov DI, et al. Stress-related menstrual disorders. Consilium Medicum. 2016;18(6):8-13. (In Russ.).

[cit6] Prior Jerilynn C. (2022). Adaptive, reversible, hypothalamic reproductive suppression: More than functional hypothalamic amenorrhea. Frontiers in Endocrinology.

[cit7] (2009). The Female Athlete Triad. Medicine & Science in Sports & Exercise.

[cit8] Morrison Amy E., Fleming Suzannah, Levy Miles J. (2020). A review of the pathophysiology of functional hypothalamic amenorrhoea in women subject to psychological stress, disordered eating, excessive exercise or a combination of these factors. Clinical Endocrinology.

[cit9] Pedreira Clarissa Carvalho, Maya Jacqueline, Misra Madhusmita (2022). Functional hypothalamic amenorrhea: Impact on bone and neuropsychiatric outcomes. Frontiers in Endocrinology.

[cit10] Bonazza Federica, Politi Giuliana, Leone Daniela, Vegni Elena, Borghi Lidia (2023). Psychological factors in functional hypothalamic amenorrhea: A systematic review and meta-analysis. Frontiers in Endocrinology.

[cit11] Bobrov A.E., Il'ina N.A. Psikhicheskie rasstroistva u zhenshchin, stradayushchikh chastymi formami vtorichnoi amenorei: nozograficheskaya struktura i psikhologicheskie markery // Rossiiskii psikhiatricheskii zhurnal. — 2022. — №5. — S. 74-83.

[cit12] Pruneti Carlo, Guidotti Sara (2022). Cognition, Behavior, Sexuality, and Autonomic Responses of Women with Hypothalamic Amenorrhea. Brain Sciences.

[cit13] Misra Madhusmita, Katzman Debra K., Estella Nara Mendes, Eddy Kamryn T., Weigel Thomas, Goldstein Mark A., Miller Karen K., Klibanski Anne (2013). Impact of Physiologic Estrogen Replacement on Anxiety Symptoms, Body Shape Perception, and Eating Attitudes in Adolescent Girls With Anorexia Nervosa. The Journal of Clinical Psychiatry.

[cit14] Baskaran Charumathi, Cunningham Brooke, Plessow Franziska, Singhal Vibha, Woolley Ryan, Ackerman Kathryn E., Slattery Meghan, Lee Hang, Lawson Elizabeth A., Eddy Kamryn, Misra Madhusmita (2017). Estrogen Replacement Improves Verbal Memory and Executive Control in Oligomenorrheic/Amenorrheic Athletes in a Randomized Controlled Trial. The Journal of Clinical Psychiatry.

[cit15] Baskaran Charumathi, Plessow Franziska, Ackerman Kathryn E., Singhal Vibha, Eddy Kamryn T., Misra Madhusmita (2017). A cross-sectional analysis of verbal memory and executive control across athletes with varying menstrual status and non-athletes. Psychiatry Research.

[cit16] Lawson Elizabeth A., Donoho Daniel, Miller Karen K., Misra Madhusmita, Meenaghan Erinne, Lydecker Janet, Wexler Tamara, Herzog David B., Klibanski Anne (2009). Hypercortisolemia Is Associated with Severity of Bone Loss and Depression in Hypothalamic Amenorrhea and Anorexia Nervosa. The Journal of Clinical Endocrinology & Metabolism.

[cit17] Fontana L., Garzia E., Marfia G., Galiano V., Miozzo M. (2022). Epigenetics of functional hypothalamic amenorrhea. Frontiers in Endocrinology.

[cit18] Bullen Beverly A., Skrinar Gary S., Beitins Inese Z., von Mering Gretchen, Turnbull Barry A., McArthur Janet W. (2010). Induction of Menstrual Disorders by Strenuous Exercise in Untrained Women. New England Journal of Medicine.

[cit19] Lazarev A.M., Bezuglov E.N., Barskova E.M., Rusanov M.O. (2021). Effects of intense running on menstrual function in semi-professional adult runners. Problemy reproduktsii.

[cit20] Bethea Cynthia L., Phu Kenny, Reddy Arubala P., Cameron Judy L. (2013). The effect of short moderate stress on the midbrain corticotropin-releasing factor system in a macaque model of functional hypothalamic amenorrhea. Fertility and Sterility.

[cit21] Centeno M.‐L., Sanchez R. L., Cameron J. L., Bethea C. L. (2007). Hypothalamic Gonadotrophin‐Releasing Hormone Expression in Female Monkeys with Different Sensitivity to Stress. Journal of Neuroendocrinology.

[cit22] Delaney Angela, Burkholder Adam B, Lavender Christopher A, Plummer Lacey, Mericq Veronica, Merino Paulina M, Quinton Richard, Lewis Katie L, Meader Brooke N, Albano Alessandro, Shaw Natalie D, Welt Corrine K, Martin Kathryn A, Seminara Stephanie B, Biesecker Leslie G, Bailey-Wilson Joan E, Hall Janet E (2020). Increased Burden of Rare Sequence Variants in GnRH-Associated Genes in Women With Hypothalamic Amenorrhea. The Journal of Clinical Endocrinology & Metabolism.

[cit23] Cangiano B, Swee DS, Quinton R, Bonomi M. Genetics of congenital hypogonadotropic hypogonadism: peculiarities and phenotype of an oligogenic disease. Hum Genet. 2021;140(1):77-111. doi: https://doi.org/10.1007/s00439-020-02147-1 32200437

[cit24] Oyola Mario G., Handa Robert J. (2017). Hypothalamic–pituitary–adrenal and hypothalamic–pituitary–gonadal axes: sex differences in regulation of stress responsivity. Stress.

[cit25] Gan Yiqun, Chen Yidi, Han Xiao, Yu Nancy Xiaonan, Wang Li (2019). Neuropeptide Y Gene × Environment Interaction Predicts Resilience and Positive Future Focus. Applied Psychology: Health and Well-Being.

[cit26] Meczekalski Blazej, Genazzani Andrea R., Genazzani Alessandro D., Warenik-Szymankiewicz Alina, Luisi Michele (2006). Clinical evaluation of patients with weight loss-related amenorrhea: Neuropeptide Y and luteinizing hormone pulsatility. Gynecological Endocrinology.

[cit27] Miranda M, Morici JF, Zanoni MB, Bekinschtein P. Brain-derived neurotrophic factor: A key molecule for memory in the healthy and the pathological brain. Front Cell Neurosci. 2019;(13):363. doi: https://doi.org/10.3389/fncel.2019.00363 PMC6692714 31440144

[cit28] Podfigurna-Stopa Agnieszka, Casarosa Elena, Luisi Michele, Czyzyk Adam, Meczekalski Blazej, Genazzani Andrea Riccardo (2013). Decreased plasma concentrations of brain-derived neurotrophic factor (BDNF) in patients with functional hypothalamic amenorrhea. Gynecological Endocrinology.

[cit29] Koysombat Kanyada, Dhillo Waljit S., Abbara Ali (2023). Assessing hypothalamic pituitary gonadal function in reproductive disorders. Clinical Science.

[cit30] Argente Jesús, Dunkel Leo, Kaiser Ursula B, Latronico Ana C, Lomniczi Alejandro, Soriano-Guillén Leandro, Tena-Sempere Manuel (2023). Molecular basis of normal and pathological puberty: from basic mechanisms to clinical implications. The Lancet Diabetes & Endocrinology.

[cit31] Teles Milena Gurgel, Bianco Suzy D.C., Brito Vinicius Nahime, Trarbach Ericka B., Kuohung Wendy, Xu Shuyun, Seminara Stephanie B., Mendonca Berenice B., Kaiser Ursula B., Latronico Ana Claudia (2008). AGPR54-Activating Mutation in a Patient with Central Precocious Puberty. New England Journal of Medicine.

[cit32] Podfigurna Agnieszka, Maciejewska-Jeske Marzena, Meczekalski Blazej, Genazzani Alessandro D. (2020). Kisspeptin and LH pulsatility in patients with functional hypothalamic amenorrhea. Endocrine.

[cit33] Meczekalski Blazej, Niwczyk Olga, Bala Gregory, Szeliga Anna (2022). Stress, kisspeptin, and functional hypothalamic amenorrhea. Current Opinion in Pharmacology.

[cit34] Jayasena Channa N., Nijher Gurjinder M. K., Chaudhri Owais B., Murphy Kevin G., Ranger Amita, Lim Adrian, Patel Daksha, Mehta Amrish, Todd Catriona, Ramachandran Radha, Salem Victoria, Stamp Gordon W., Donaldson Mandy, Ghatei Mohammad A., Bloom Stephen R., Dhillo Waljit S. (2009). Subcutaneous Injection of Kisspeptin-54 Acutely Stimulates Gonadotropin Secretion in Women with Hypothalamic Amenorrhea, But Chronic Administration Causes Tachyphylaxis. The Journal of Clinical Endocrinology & Metabolism.

[cit35] Jayasena C N, Nijher G M K, Abbara A, Murphy K G, Lim A, Patel D, Mehta A, Todd C, Donaldson M, Trew G H, Ghatei M A, Bloom S R, Dhillo W S (2010). Twice-Weekly Administration of Kisspeptin-54 for 8 Weeks Stimulates Release of Reproductive Hormones in Women With Hypothalamic Amenorrhea. Clinical Pharmacology & Therapeutics.

[cit36] Jayasena Channa N., Abbara Ali, Veldhuis Johannes D., Comninos Alexander N., Ratnasabapathy Risheka, De Silva Akila, Nijher Gurjinder M. K., Ganiyu-Dada Zainab, Mehta Amrish, Todd Catriona, Ghatei Mohammad A., Bloom Stephen R., Dhillo Waljit S. (2014). Increasing LH Pulsatility in Women With Hypothalamic Amenorrhoea Using Intravenous Infusion of Kisspeptin-54. The Journal of Clinical Endocrinology & Metabolism.

[cit37] Abbara Ali, Eng Pei Chia, Phylactou Maria, Clarke Sophie A., Richardson Rachel, Sykes Charlene M., Phumsatitpong Chayarndorn, Mills Edouard, Modi Manish, Izzi-Engbeaya Chioma, Papadopoulou Debbie, Purugganan Kate, Jayasena Channa N., Webber Lisa, Salim Rehan, Owen Bryn, Bech Paul, Comninos Alexander N., McArdle Craig A., Voliotis Margaritis, Tsaneva-Atanasova Krasimira, Moenter Suzanne, Hanyaloglu Aylin, Dhillo Waljit S. (2020). Kisspeptin receptor agonist has therapeutic potential for female reproductive disorders. Journal of Clinical Investigation.

[cit38] Bondarenko Bondarenko K.R., Kazantseva Kazantseva V.D., Dobrokhotova Yu Dobrokhotova Yu.E. (2022). Functional hypothalamic amenorrhea in clinical practice: medical and diagnostic features. Akusherstvo i ginekologiia.

[cit39] Padda Jaskamal, Khalid Khizer, Hitawala Gazala, Batra Nitya, Pokhriyal Sindhu, Mohan Ayushi, Zubair Ujala, Cooper Ayden Charlene, Jean-Charles Gutteridge (2021). Depression and Its Effect on the Menstrual Cycle. Cureus.

[cit40] Chernukha Chernukha G.E., Gusev Gusev D.V., Tabeeva Tabeeva G.I., Prilutskaya Prilutskaya V.Yu. (2018). Current principles of therapy for functional hypothalamic amenorrhea. Akusherstvo i ginekologiia.

[cit41] (2004). Revised 2003 consensus on diagnostic criteria and long-term health risks related to polycystic ovary syndrome. Fertility and Sterility.

[cit42] Zaks Nina, Batuure Anita, Lin Emma, Rommel Anna-Sophie, Reichenberg Abraham, Grice Dorothy, Bergink Veerle, Fox Nathan S., Mahjani Behrang, Janecka Magdalena (2023). Association Between Mental Health and Reproductive System Disorders in Women. JAMA Network Open.

[cit43] Ушакова В. М., Морозова А. Ю., Резник А. М., Костюк Г. П., Чехонин В. П. (2020). Молекулярно-биологические аспекты депрессивных состояний: современный взгляд на проблему. Молекулярная биология.

[cit44] Hu Runan, Geng Yuli, Huang Yanjing, Liu Zhuo, Li Fan, Dong Haoxu, Ma Wenwen, Song Kunkun, Zhang Mingmin, Zhang Zhuo, Song Yufan (2023). New insights into the interaction between polycystic ovary syndrome and psychiatric disorders: A narrative review. International Journal of Gynecology & Obstetrics.

[cit45] Wallen Kim (2005). Hormonal influences on sexually differentiated behavior in nonhuman primates. Frontiers in Neuroendocrinology.

[cit46] Moore AM, Campbell RE. Polycystic ovary syndrome: Understanding the role of the brain. Front Neuroendocrinol. 2017;46(5):1-14. doi: https://doi.org/10.1016/j.yfrne.2017.05.002 28551304

[cit47] Teede Helena J., Tay Chau Thien, Laven Joop, Dokras Anuja, Moran Lisa J., Piltonen Terhi T., Costello Michael F., Boivin Jacky, M. Redman Leanne, A. Boyle Jacqueline, Norman Robert.J., Mousa Aya, Joham Anju E., Arlt Wiebke, Azziz Ricardo, Balen Adam, Bedson Lisa, Berry Lorna, Boivin Jacky, Boyle Jacqueline, Brennan Leah, Brown Wendy, Burgert Tania, Busby Maureen, Ee Carolyn, Garad Rhonda M., Gibson-Helm Melanie, Harrison Cheryce, Hart Roger, Hopkins Kim, Hirschberg Angelica Lindén, Ho Tuong, Hoeger Kathleen, Jordan Cailin, Legro Richard S., Li Rong, Lujan Marla, Ma Ronald, Mansfield Darren, Marsh Kate, Mocanu Edgar, Mol Ben, Mormon Rachel, Norman Robert, Oberfield Sharon, Patel Malika, Pattuwage Loyal, Peña Alexia, Redman Leanne, Rombauts Luk, Romualdi Daniela, Shah Duru, Spritzer Poli Mara, Stener-Victorin Elisabet, Tehrani Fahimeh Ramezani, Thangaratinam Shakila, Thondan Mala, Vanky Eszter, Wijeyaratne Chandrika, Witchel Selma, Yang Dongzi, Yildiz Bulent, Alesi Simon, Alur-Gupta Snigdha, Avery Jodie, Khomami Mahnaz Bahri, Benham Jamie, Bidstrup Hugh, Chua Su Jen, Cooney Laura, Coster Thisara, Ee Carolyn, Fitz Victoria, Flanagan Madeline, Forslund Maria, Jiskoot Geranne, Kazemi Maryam, Kempegowda Punith, Louwers Yvonne, Lujan Marla, Melin Johanna, Melson Eka, Mengistu Yitayeh Belsti, Naderpoor Negar, Neven Adriana, Pastoor Hester, Rocha Thais, Sabag Angelo, Subramanian Anuradhaa, Tan Katrina (2023). Recommendations from the 2023 International Evidence-based Guideline for the Assessment and Management of Polycystic Ovary Syndrome. Fertility and Sterility.

[cit48] Rempert Ashley N., Sarria Isabella, Standeven Lindsay R., Nylander Elizabeth, Segars James, Singh Bhuchitra (2023). A Systematic Review of the Psychosocial Impact of Polycystic Ovarian Syndrome Before and After Treatment. Reproductive Sciences.

[cit49] Adamczak Agnieszka, Płotek Włodzimierz, Głowińska Aleksandra, Sobol Małgorzata, Wysocka Ewa, Polak Grzegorz, Dymanowska-Dyjak Izabela, Spaczyńska Julia, Adamczak Łukasz, Banaszewska Beata (2023). Time Perspective as a Mediator of Depressive Symptoms in Patients with Polycystic Ovary Syndrome. Healthcare.

[cit50] Brutocao Claire, Zaiem Feras, Alsawas Mouaz, Morrow Allison S., Murad M. Hassan, Javed Asma (2018). Psychiatric disorders in women with polycystic ovary syndrome: a systematic review and meta-analysis. Endocrine.

[cit51] Majidzadeh Sheida, Mirghafourvand Mojgan, Farvareshi Mahmoud, Yavarikia Parisa (2023). The effect of cognitive behavioral therapy on depression and anxiety of women with polycystic ovary syndrome: a randomized controlled trial. BMC Psychiatry.

[cit52] Dema Hana, Videtič Paska Alja, Kouter Katarina, Katrašnik Mojca, Jensterle Mojca, Janež Andrej, Oblak Aleš, Škodlar Borut, Bon Jurij (2023). Effects of Mindfulness-Based Therapy on Clinical Symptoms and DNA Methylation in Patients with Polycystic Ovary Syndrome and High Metabolic Risk. Current Issues in Molecular Biology.

[cit53] Santos Isis K., Pichini Gabriel S., Daniel d. Ferreira Carlindo, Dantas Pedro B., Browne Rodrigo A. V., de Queiros Victor, Soares Gustavo M., Gonçalves Ana K., Cabral Breno G., Maranhão Tecia Maria O., Dantas Paulo Moreira S. (2022). Effects of high-intensity interval training in combination with detraining on mental health in women with polycystic ovary syndrome: A randomized controlled trial. Frontiers in Physiology.

[cit54] Patten Rhiannon K., McIlvenna Luke C., Moreno-Asso Alba, Hiam Danielle, Stepto Nigel K., Rosenbaum Simon, Parker Alexandra G. (2023). Efficacy of high-intensity interval training for improving mental health and health-related quality of life in women with polycystic ovary syndrome. Scientific Reports.

[cit55] Greenwood Eleni A., Pasch Lauri A., Cedars Marcelle I., Legro Richard S., Eisenberg Esther, Huddleston Heather G. (2018). Insulin resistance is associated with depression risk in polycystic ovary syndrome. Fertility and Sterility.

[cit56] Irandoust Khadijeh, Taheri Morteza (2018). Effect of a High Intensity Interval Training (HIIT) on Serotonin and Cortisol Levels in Obese Women With Sleep Disorders. Women’s Health Bulletin.

[cit57] Li Xinqiang, Wang Shufen, Zhang Li, Zhang Lei, Liu Jiali, Luo Haoshu, Gou Kemian, Cui Sheng (2017). Amitriptyline plays important roles in modifying the ovarian morphology and improving its functions in rats with estradiol valerate-induced polycystic ovary. Archives of Pharmacal Research.

[cit58] Alkan Işınsu, Kaplan Süleyman (2023). An investigation of the potential effects of amitriptyline on polycystic ovary syndrome induced by estradiol valerate. Histochemistry and Cell Biology.

[cit59] Rebar Robert W., Keator Christopher S. (2020). Expanding our knowledge of premature ovarian insufficiency. Fertility and Sterility.

[cit60] Li M., Zhu Y., Wei J., Chen L., Chen S., Lai D. (2022). The global prevalence of premature ovarian insufficiency: a systematic review and meta-analysis. Climacteric.

[cit61] Hu Mengwen, Yeh Yu-Han, Munakata Yasuhisa, Abe Hironori, Sakashita Akihiko, Maezawa So, Vidal Miguel, Koseki Haruhiko, Hunter Neil, Schultz Richard M., Namekawa Satoshi H. (2022). PRC1-mediated epigenetic programming is required to generate the ovarian reserve. Nature Communications.

[cit62] Xi Dan, Chen Biyin, Tao Hui, Xu Yunxiang, Chen Guizhen (2023). The risk of depressive and anxiety symptoms in women with premature ovarian insufficiency: a systematic review and meta-analysis. Archives of Women's Mental Health.

[cit63] Ventura June L., Fitzgerald O. Ray, Koziol Deloris E., Covington Sharon N., Vanderhoof Vien H., Calis Karim A., Nelson Lawrence M. (2007). Functional well-being is positively correlated with spiritual well-being in women who have spontaneous premature ovarian failure. Fertility and Sterility.

[cit64] Chu Kun, Wang Yining, He Yi, Tang Yunxiang, Gu Jiayi, Wu Shuang, Zhang Honghong, Sun Ningxia, Li Ziyuan, Zhang Qing, Li Wen (2020). The psychosocial impact of premature ovarian insufficiency on male partners and their perceptions of the disease. Psychology, Health & Medicine.

[cit65] Gordon Jennifer L., Eisenlohr-Moul Tory A., Rubinow David R., Schrubbe Leah, Girdler Susan S. (2016). Naturally Occurring Changes in Estradiol Concentrations in the Menopause Transition Predict Morning Cortisol and Negative Mood in Perimenopausal Depression. Clinical Psychological Science.

[cit66] Liu Jian, Huang Xunchun, Cao Xiaojing, Feng Xuan, Wang Xiaoyun (2019). Serum biomarker analysis in patients with premature ovarian insufficiency. Cytokine.

[cit67] Yusuf Lamia (2016). Depression, anxiety and stress among female patients of infertility; A case control study. Pakistan Journal of Medical Sciences.

[cit68] Golezar Samira, Keshavarz Zohreh, Ramezani Tehrani Fahime, Ebadi Abbas (2020). An exploration of factors affecting the quality of life of women with primary ovarian insufficiency: a qualitative study. BMC Women's Health.

[cit69] Zefferino Roberto, Di Gioia Sante, Conese Massimo (2020). Molecular links between endocrine, nervous and immune system during chronic stress. Brain and Behavior.

